# Effects of anger and trigger identity on triggered displaced aggression among college students: based on the “kicking the barking dog effect”

**DOI:** 10.1186/s40359-024-02118-5

**Published:** 2024-11-08

**Authors:** Shen Liu, Wenxiu Li, Xinwei Hong, Minghua Song, Feng Liu, Zhibin Guo, Lin Zhang

**Affiliations:** 1 Department of Psychology, School of Humanities and Social Sciences, Anhui Agricultural University, No. 130 Changjiang Road(W), Shushan District, Hefei, Anhui 230036 China; 2 Department of Psychology, Renmin University of China, Beijing, China; 3 Department and Institute of Psychology, Ningbo University, No. 616 Fenghua Road, Jiangbei District, Ningbo, Zhejiang 315211 China; 4 Mental Health Education Guidance Center, Huzhou University, Huzhou, Zhejiang China; 5 Xiamen University Tan Kan Kee College, Zhangzhou, Fujian China

**Keywords:** Triggered displaced aggression, Anger, Trigger identity, Hostile attribution, College students

## Abstract

**Background:**

Drawing on the “kicking the barking dog effect”, this study investigated the individual and group-level mechanisms underlying triggered displaced aggression (TDA).

**Methods:**

Three experiments were conducted to investigate the effects of anger and hostile attribution on TDA, examining these factors at both the individual and group levels. The preliminary experiment investigated how emotions affect subsequent cognition at an individual level with the aim of understanding the underlying generative process of TDA. Experiment 1 explored the relationship between anger, hostile attribution, and subsequent aggressive behaviors at an individual level, while also discussing the underlying generative process of TDA. Experiment 2 investigated the relationship between anger, hostile attribution, and subsequent aggressive behaviors from a group-level perspective, while also delving into the impact of trigger identity on the underlying generative process of TDA.

**Results:**

When individuals were exposed to both a provocation and a subsequent triggering situation, they demonstrated stronger hostile attribution and displayed more aggressive behavior directed toward the trigger. This study found that hostile attribution played a complete mediating role in the influence of anger on TDA, while the triggering situation itself had a moderating role. In the presence of triggering situations, individuals exhibited stronger hostile attribution as their level of anger increased. In the absence of a triggering situation, changes in anger level did not have a significant effect. In the presence of a triggering situation, the identity of the trigger played a moderating role in the process of “anger → hostile attribution → TDA”. When the trigger belonged to an in-group, individuals exhibited stronger hostile attribution toward the out-group and subsequently displayed greater TDA.

**Conclusion:**

These findings expand the applicability of the “kicking the barking dog effect” and offer suggestions for controlling the escalation of intergroup conflicts.

**Supplementary Information:**

The online version contains supplementary material available at 10.1186/s40359-024-02118-5.

## Introduction

“A man who was severely berated by his boss at work chose to submit to the humiliation, fearing the loss of his job. When he arrived home and was greeted by his dog barking at the door, he did not treat his dog as friendly as usual. Instead, he forcefully kicked the dog away” [1]. This example illustrates the “kicking the barking dog effect,” where the strong provocation from his boss contrasted with the relatively minor trigger of the barking dog led the man to displace his frustrations onto the more vulnerable target - his dog. This displacement of aggression from the original source to a substitute target is the essence of triggered displaced aggression (TDA) [2, 3], demonstrated in this scenario. TDA refers to a reaction where an individual, unable to directly respond to an initial, strong provocation, subsequently performs aggressive behaviors that do not match the minor conflict at hand when provoked by a much weaker trigger in another situation.

TDA can manifest in many contexts, including instances of domestic violence, animal abuse, and child abuse [4, 5]. It is critical to recognize that TDA can also contribute to the onset and intensification of group conflicts [6, 7], with potential repercussions for social stability. The far-reaching implications of this psychological phenomenon emphasize the importance of gaining a deeper understanding of and addressing TDA. Existing research on TDA has predominantly concentrated on its external influencing factors, such as the attributes of the trigger and the resemblance between the trigger and the substitute target. For instance, Norman Miller and colleagues found that aggressive reactions to minor triggering provocations could be disproportionately intense when they followed a moderately strong provocation that precluded retaliation [2]. Albert Reijntjes and colleagues found that displaced aggression was evident only when the negative feedback was potent, and participants were unable to retaliate directly. Higher levels of callousness specifically correlated with increased displaced (but not direct) aggression. However, the amplifying impacts of callousness manifested solely when the negative feedback was powerful [8]. Additionally, Albert Reijntjes and colleagues observed that in reaction to negative feedback, participants disproportionately displaced aggression disproportionally onto innocent Moroccan out-group targets. This outcome was not influenced by ethnic prejudice; regardless of the ethnicity of the target, participants with more negative attitudes toward Moroccans exhibited elevated levels of aggression in both conditions [9]. Moreover, Eduardo A. Vasquez and colleagues found that inhibitory cues diminished displaced aggression under conditions of low cognitive load. However, when participants in the inhibitory cue condition experienced cognitive load, aggression escalated, indicating that mental busyness impeded the complete utilization of inhibitory information [10]. This body of work offers important insights into the situational factors that drive displaced aggression. However, these studies have given little attention to the internal process of TDA, specifically the psychological mechanisms that lead individuals to displace their aggression in this manner. This study was grounded in the “kicking the barking dog effect” theory and explored the mechanism of TDA from both individual and group perspectives. This study offers valuable insights that could aid in preventing and mitigating the escalation of violent group conflicts. By adopting a more comprehensive approach to understanding TDA, this study provides important references for addressing this complex social issue.

According to the general aggression model, an individual’s emotion and cognition, as internal factors, can influence the expression of aggressive behaviors [11]. As a distinctive form of aggressive behavior, TDA can also be particularly influenced by negative emotions, such as anger and depression, which are often considered predictors of aggressive behaviors [12–17]. Importantly, the provocative anger stemming from initial strong provocations also plays a key role in TDA [2, 5, 18]. The ego depletion theory posits that the total amount of self-control resources an individual possesses is limited. As a result, following a period of activities that demand the use of self-control resources, the individual’s self-control ability becomes depleted, potentially affecting the performance of subsequent tasks that also require self-control [19, 20]. In simpler terms, when individuals face a provocative situation that triggers anger, they may try to regulate their words and actions through self-control in order to adhere to social norms. Subjective cognition, emotion, and other factors have been shown to lead to ego depletion [21], which may result in individuals engaging in immoral and risk-taking behaviors in subsequent triggering situations [22]. Nevertheless, a time interval exists between the onset of anger sparked by the initial provocation and the subsequent trigger that leads to displaced aggression towards another. When this interval is brief, the initial anger may not have sufficient time to subside, and the level of physiological arousal could impact subsequent aggressive behaviors. However, when this interval is long, the anger and accompanying physiological arousal may last for only 10–20 min without any further processing. The individual’s rumination on anger plays a role in maintaining it, which can impact subsequent aggressive behavior. It also leads to the generation of a series of cognitions related to aggression. The accessibility of aggressive cognition leads individuals to generate an attribution bias in the triggering situation [1]. With this in mind, this study attempted to investigate how anger influences the cognitive pathway of TDA.

As per the cognitive-neoassociation theory of aggression, the anger triggered by a provocative situation can activate the memory network linked to aggression. This activation may lead the individual to form a hostile explanation for the subsequent mild triggering situation, ultimately resulting in aggressive behaviors toward the target [14]. Likewise, other studies have shown that anger and cognition related to aggression can be triggered by the provocative situation, subsequently leading to the individual’s attribution bias toward the mild stimulus in the subsequent triggering situation, specifically a hostile attribution [23]. Anger, in comparison to sadness and neutral emotions, has been found to prompt individuals to make more hostile inferences [24, 25]. Prior researches have shown a positive correlation between hostile attribution and aggressive behaviors [26, 27], and has indicated that hostile attribution plays a mediating role in the relationship between provocative anger and aggressive behaviors [23]. Barlett and Anderson proposed that individuals with high levels of hostile attribution tend to focus on hostile cues, ultimately shaping a hostile model of social information [28]. The social information processing model suggests that hostile attribution leads individuals to process and interpret others’ behavioral intentions in a hostile mode. Even if the actual intention of others is harmless, it is easier to make hostile assessments, which in turn can trigger aggressive behaviors toward others [29–31]. As a result, the anger induced by the previous provocation will influence the individual’s cognition of the subsequent triggering situation, ultimately leading to TDA. Taking this into account, this study posited the following hypothesis: hostile attribution plays a mediating role between anger and TDA (*H*_1_).

In TDA, individuals initially encounter a highly provocative situation, leading to a surge in anger. However, the target of attack is not the individual who provokes it, but rather switches to the trigger. This target shift causes the impact of TDA to spread from the individual to the group, offering a new perspective on the emergence and escalation of group conflicts [32]. The majority of the individual’s activities occur within a specific group situation. In recent years, the rise in social conflicts and the rapid changes in people’s mindset have heightened the risk of intergroup conflicts [33]. Among the manifestations of intergroup conflict, intergroup violent conflicts (such as violent disputes between different families and terrorist events involving different nationalities and religions) are particularly noteworthy. This type of conflict not only leads to serious adverse effects for both sides of the dispute, but also contributes to heightened social instability. However, intergroup violent conflict in real life often involves not a direct violent confrontation between two complete groups, but rather a type of conflict stemming from violence and escalating attacks between members of the two groups [34, 35]. However, existing aggression theories may struggle to explain the process of intergroup conflict spreading from individuals to groups. For instance, while the general aggression model illustrates how a social encounter can impact an individual’s internal state and decision-making ability, ultimately leading to aggressive behavior, it may not provide an explanation for how an attack between individuals extends to their respective groups [11]. According to the group identity theory of the social identity model and the intergroup social relations model, each group member exhibits an in-group preference. The higher the level of identification with the in-group, the more likely there is to be feelings of disgust and belittlement toward the out-group. The outgroup identity is seen as a threat to the group and is considered a source of hostile and competitive motivation, leading to the adoption defensive coping styles, such as aggressive behavior [36–38]. In other words, when the target and the provocator belong to the same out-group, the more physically similar and closely related they are, the stronger the satisfaction derived from the attack [39]. Previous studies have also found that the characteristics of an aggressive target, such as the identity of the provocateur, may affect TDA [5, 39]. For instance, when an individual is provoked by a negative evaluation, compared with the in-group, the individual displays stronger displaced aggression when the target is a member of an outgroup. When the individual is not provoked, there is no significant difference in displaced aggression toward in-group and out-group targets [8, 9]. Furthermore, hostile attribution is significantly positively correlated with trigger identity. The individual is more inclined to process and interpret the behavioral intentions in a hostile manner when the trigger is an out-group member, as opposed to an in-group member [2]. Intergroup threat theory also indicates that the presence of an out-group poses a certain threat to the in-group, leading to the arousal of negative emotion (such as anger) among in-group members toward the out-group. Additionally, as the level of intergroup threat rises, negative emotions will increase, impacting the in-group’s hostile attribution toward the out-group members’ behavioral intentions and potentially triggering intergroup aggressive behavior [40–42]. Previous studies have shown that the more positive the contact between individuals and the out-group, the less likely the individual is to behave aggressively toward the out-group. This variation is indirectly produced through the decrease in threat perception among groups [43]. In other words, when individuals are provoked to feel anger by a trigger, they are more likely to generate hostile explanations for an out-group trigger compared to an in-group trigger in a subsequent weak triggering situation. The hostile explanation influences the intensity of the attack on the trigger. Therefore, this study proposed the following hypothesis: the relationship between anger and TDA is moderated by trigger identity, and it moderates the first half path and direct path of the mediating process of “anger → hostile attribution → TDA” (*H*_2_).

In summary, this study was based on the “kicking barking dog effect” theory. Three experiments were conducted to investigate the effects of anger and hostile attribution in TDA within the framework of individuals and groups. The preliminary experiment examined the effects of emotions on subsequent cognition within the individual framework to investigate the process of generating TDA. Experiment 1 examined the relationship between anger, hostile attribution, and subsequent aggressive behaviors within the individual framework, and discussed the process of generating TDA. Experiment 2 examined the relationship between anger, hostile attribution, and subsequent aggressive behaviors within the group framework, and further explored the effect of trigger identity in the process of generating TDA. As such, this study explored the mechanism of TDA while also preliminarily exploring the escalation of intergroup violent conflict from the perspective of trigger identity.

## Preliminary experiment: The effect of anger on hostile attribution and aggressive intention

### Participants

The participants included 50 undergraduate students (31 males and 19 females) from a university who were selected at random. Out of these, three did not complete the experiment and were therefore eliminated from this study. The final number of participants was 47 (30 males and 17 females), with an age range of 18–27 years old (average age = 23.11, *SD* = 2.25). Participants were randomly assigned to either the experimental group (*n* = 24) or the control group (*n* = 23). This study was approved by the Ethics Committee of Anhui Agricultural University in accordance with the ethical principles of the Declaration of Helsinki. All participants provided written informed consent prior to this study, and after completing this study, they received monetary compensation (the same procedure was applied to Experiments 1 and 2).

## Materials

### Anger

To measure participants’ anger, we utilized the Emotional State Self-Rating scale by Peng et al. [44]. On seven items, participants indicated the extent to which they experienced emotions on the scale, such as “hate”. The original 5-point scoring in the scale was modified to a 7-point scoring (1="none at all”; 7="extremely strong”) to ensure consistency of the scores. Higher scores indicated stronger anger. The internal consistency coefficient for the scale was calculated as 0.86.

### Hostile attribution

Following Gagnon et al. [45], six ambiguous situations were selected, each containing two hostile and two non-hostile explanations. These situations could be interpreted as either hostile or non-hostile. A 7-point scoring system was adopted, ranging from 1 (impossible at all) to 7 (very possible). Participants’ hostile attribution in the ambiguous situations was measured based on the score given to the hostile explanation. The higher the score, the stronger the hostile attribution. The internal consistency coefficient for the explanation of hostility in all situations was calculated as 0.77.

### Aggressive intention

To measure the participants’ aggressive intention toward individuals in ambiguous situations, direct questions were utilized, such as “How much do you want to be angry with these two classmates?” A 7-point scoring system was employed, with ratings ranging from 1="not at all” to 7="want a lot”. Higher scores indicated a stronger aggressive intention. Previous studies have shown that direct inquiry can establish a coherence with the aforementioned hostile attribution, helping to maintain consistency between the former and the latter target. As a result, it is more effective than using a single aggressive questionnaire or scale [3].

### Anger priming materials

Based on the work of Sjöström and Gollwitzer, the recall method was utilized to manipulate both anger-inducing situations and general situations [39]. In the anger-inducing situation, participants were asked to recall a recent experience that had generated anger. This experience was chosen specifically because it had the potential to induce strong anger at the present moment. In the general situation, participants were asked to recall the recent description of a product or the explanation of a word in a book. In both situations, participants were asked to write down the time, place, and general description of the experience they could recall. Detailed materials can be found in the Supporting Information.

### Experimental design and procedures

A single-factor experimental design (situation type: anger-inducing, general) was adopted, with the dependent variables being the individual’s scores of hostile attribution and aggressive intention. First, all participants completed the Emotional State Self-Rating scale as the pre-test, followed by the anger-inducing situation (the experimental group) and the general situation (the control group). Then, the Emotional State Self-Rating scale and measures of hostile attribution and aggressive intention were administered as the post-test assessment. The pre-test, experimental and control situation, and post-test were all conducted on the same day. After the experiment, to reassure participants, the purpose of the experiment was explained to them.

## Results

First, the validity of the provoked anger manipulation was assessed. The paired sample *t*-test revealed that in the experimental group, the post-test anger scores (*M* = 3.71, *SD* = 1.68) were significantly higher than the pre-test scores (*M* = 1.58, *SD* = 0.97; *t*_23_=–7.47, *p* < 0.001, *d* = 1.39). However, in the control group, there was no significant difference between the pre-test (*M* = 1.70, *SD* = 1.26) and post-test (*M* = 1.52, *SD* = 0.99) anger scores (*t*_22_ = 0.30, *p* = 0.730). Additionally, there was no significant difference between the experimental group and the control group in the pre-test of anger (*t*_45_ = 0.34, *p* = 0.730), indicating that the anger-inducing manipulation was effective.

Subsequently, hostile attribution and aggressive intention were used as dependent variables in the analysis of variance by a 2 (sex: male, female)×2 (situation type: provoked, general) between-group design. The results indicated that for hostile attribution, the main effect of the situation type was significant (*F*(1, 43) = 14.18, *p* < 0.001, η2 *p* = 0.251), with the experimental group demonstrating a stronger degree of hostile attribution than the control group. The main effect of sex (*F*(1, 43) = 0.16, *p* = 0.690) and the interaction (*F*(1, 43) = 1.25, *p* = 0.270) were not significant. For aggression, situation type (*F*(1, 43) = 1.34, *p* = 0.250), sex (*F*(1, 43) = 2.54, *p* = 0.120), and the interaction (*F*(1, 43) = 0.04, *p* = 0.840) were not significant.

### Experiment 1: “Kicking the barking dog effect”: The roles of anger and hostile attribution

#### Participants

A total of 90 undergraduate students, consisting of 38 males and 52 females, were randomly selected from a university. However, five of these participants either did not complete the experiment or guessed its purpose and were subsequently eliminated. As a result, the final number of participants was 85, comprising 35 males and 50 females, with ages ranging from 18 to 25 years (average age = 19.36, *SD* = 1.30).

### Materials

#### Anger

The same as the preliminary experiment.

### Hostile attribution

The Hostile Attribution Bias Scale by Topalli and O’Neal, consisting of five items [46], was utilized in this study. A 7-point scoring system was adopted, ranging from 1="completely inconsistent” to 7="completely consistent”, with higher scores indicating a stronger hostile attribution. The scale demonstrated an internal consistency coefficient of 0.88 in this study.

### Aggression

We followed Reijntjes et al.‘s approach, which suggests that a practical method to gauge an individual’s aggression towards others is by assessing the level of reward received by others [8, 9]. In this study, the reward level served as a representation aggression, with seven grades ranging from the least to the most reward. A lower reward level indicated stronger aggression. Detailed materials can be found in the Supporting Information.

### Experimental design

This study employed a 2 (provocation: yes, no)×2 (trigger: yes, no) between-group design. The dependent variables included measures of anger, hostile attribution, and the score of aggression. In this context, anger pertains to the difference in the level of anger between the pre-test and post-test, which is collectively termed as “provocative emotion” for ease of reference. Participants were allocated to four conditions: the provocative and triggering group (20 participants), the provocative and non-triggering group (18 participants), the non-provocative and triggering group (21 participants), and the non-provocative and non-triggering group (26 participants).

### Situation manipulation

Based on the classic experimental paradigm of TDA, participants received varied types of feedback designed to manipulate the preceding provocative situation and the subsequent triggering situation [3, 18].

### Provocative situation

Participants were permitted to solve a relatively difficult problem within a specified time frame, after which the experimental assistant provided them with feedback and manipulated the provocative situation based on the content of the feedback. In the provocative situation, participants received feedback such as “The answer you wrote is too groundless! I thought the people who came to participate in the experiment were at least on the same level…but your answer…ho ho…”. In contrast, in the non-provocative situation, the feedback given was “The total score is 10, you got 6.7 points, which is above the average level.”

### Triggering situation

To implement the triggering event, the participants were asked to list as many qualities an astronaut has as they could think of within a limited time and explain their reasons. In the triggering situation, participants received scores of 1, 2, 3, 1, 3, 2 in each aspect and total scores, along with the feedback “In my opinion, the completion is not good, I think that, as a person who has the ability to think independently, you should perform better.” On the other hand, in the non-triggering situation, participants received corresponding scores of 6, 5, 6, 5, 5, 5, and they were given the feedback “It’s okay to complete it in a limited time.”

### Procedure

Initially, the participants were briefed that this study focused on problem-solving abilities, and they were required to complete basic information and emotional pre-test questionnaires. Then, the participants collaborated with experimental assistant A to solve the initial problem. Following the problem-solving task, participants received feedback from experimental assistant A based on the assigned situation. The participants were unable to directly challenge the feedback provided by experimental assistant A through computer-based communication. After receiving the feedback, the participants proceeded to complete the emotional post-test. Later, the participants collaborated with experimental assistant B to solve another problem, following which they received feedback from experimental assistant B based on their assigned situation. Following the feedback, participants completed the measurement of hostile attribution towards experimental assistant B and determined the reward level that experimental assistant B should receive, which served as the measurement of aggression. Finally, the experimenter asked the participants if they had guessed the true purpose of the experiment, explained the experiment’s content, offered psychological counseling to soothe their emotions, and provided them with the corresponding monetary compensation.

## Results

Initially, the validity of the induced anger manipulation was assessed. The paired sample *t*-test revealed a significant increase in post-test (*M* = 3.58, *SD* = 1.64) compared to pre-test (*M* = 1.05, *SD* = 0.32; *t*_37_=–9.59, *p* < 0.001, *d* = 1.64) in the provocative situation group. Conversely, there was no significant difference found between the pre-test (*M* = 1.06, *SD* = 0.32) and the post-test (*M* = 1.15, *SD* = 0.42) in the non-provocative situation group (*t*_46_=–1.66, *p* = 0.100). Additionally, no significant difference existed between the two groups of participants in the anger pre-test (*t*_83_ = 0.16, *p* = 0.870), indicating that the operation to provoke anger in the experiment was indeed effective.

### Differences of hostile attribution and aggression in different situations

The results of variance analysis are shown in Table 1.

**Table 1:** Participants’ hostile attribution and aggression in different types of situation (*M ± SD*)

	Situation type	Hostile attribution	Aggression
No Provocation	no trigger (*n* = 26)	9.77 ± 3.72	2.19 ± 0.90
	trigger (*n* = 21)	10.76 ± 4.10	2.38 ± 1.16
Provocation	no trigger (*n* = 18)	11.44 ± 3.18	2.00 ± 1.03
	trigger (*n* = 20)	21.10 ± 4.81	4.20 ± 1.01

**Fig. 1:**
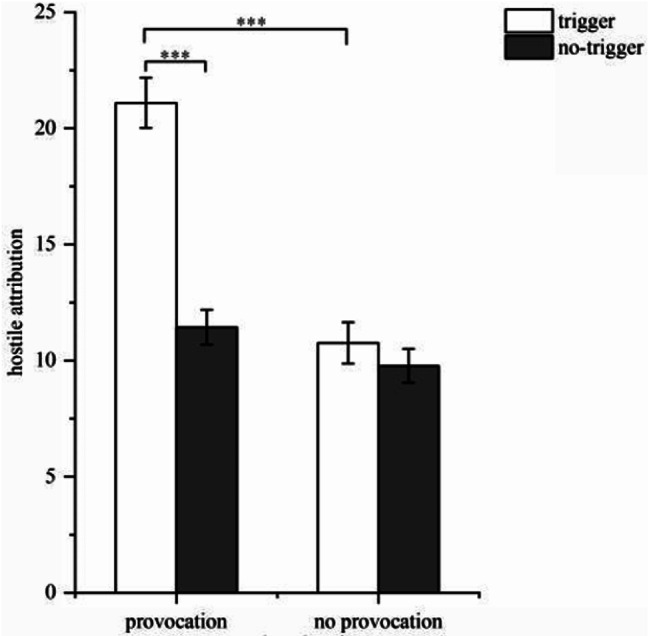
Hostile attribution towards the trigger (*Note*: ^***^p < 0.001)

The results of the variance analysis revealed that for hostile attribution, the main effect of the provocative situation was significant (*F*(1, 81) = 47.12, *p* < 0.001, η2 *p* = 0.371), with hostile attributions in the provocative situation being significantly higher than in the non-provocative situation. Similarly, the triggering situation had a significant main effect (*F*(1, 81) = 37.02, *p* < 0.001, η2 *p* = 0.313), with significantly higher hostile attributions in the triggering situation compared to the non-triggering situation. The interaction was also found to be significant (*F*(1, 81) = 24.50, *p* < 0.001, η2 *p* = 0.230). A simple effect analysis indicated a significant difference (*p* < 0.001) in hostile attribution between the presence and absence of trigger in the provocative situation, where participants in the triggering situation demonstrated stronger hostile attributions. In the triggering situation, the difference in hostile attribution between the presence and absence of provocation was also significant (*p* < 0.001), with participants in the provocative situation showing stronger hostile attributions. Conversely, in the non-provocative situation (*p* < 0.05) and non-triggering situation (*p* = 0.180), there were no significant differences. These findings suggest that participants exhibited stronger hostile attributions when exposed to both the provocative and triggering situations (see Fig. 1).

**Fig. 2:**
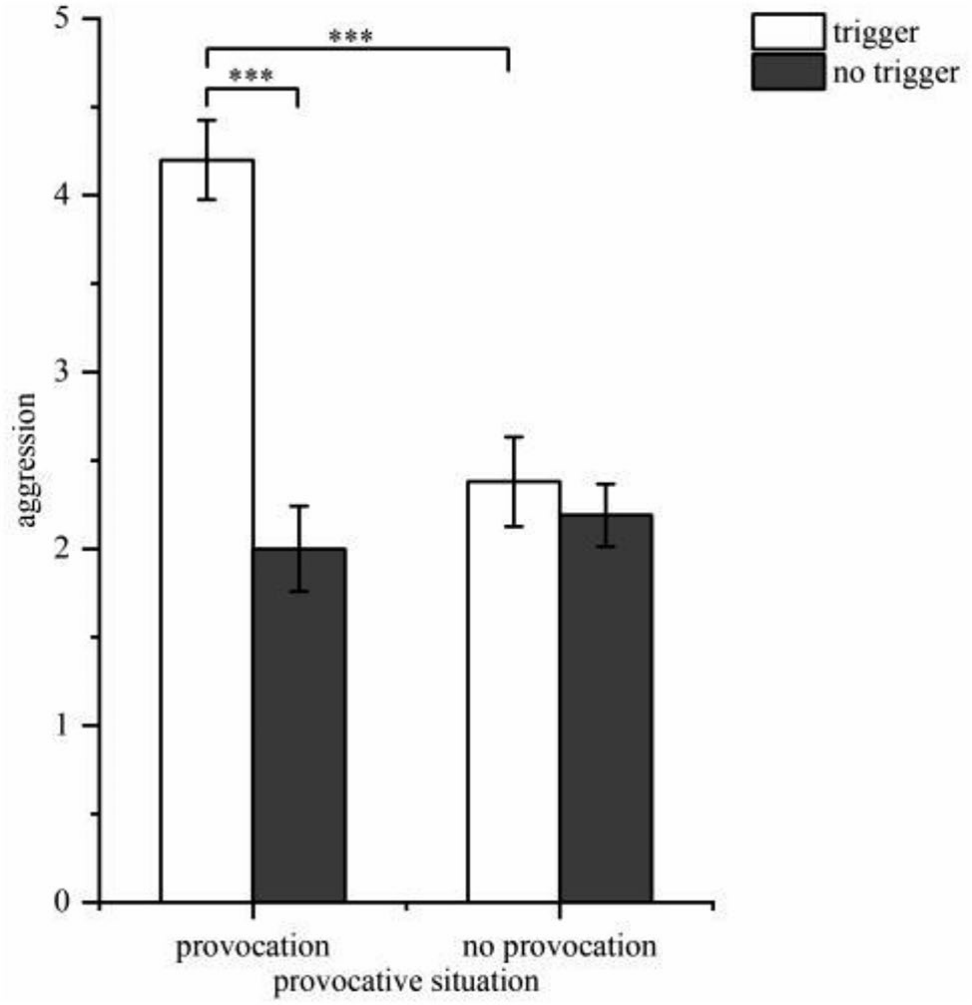
Aggression toward the trigger (*Note*: ^***^p < 0.001)

The analysis of aggression showed that the main effect of the provocative situation was significant (*F*(1, 81) = 13.29, *p* < 0.001, η2 *p* = 0.142), with aggression in the provocative situation being significantly higher than in the non-provocative situation. Similarly, the main effect of the triggering situation was significant (*F*(1, 81) = 28.64, *p* < 0.001, η2 *p* = 0.261), with aggression in the triggering situation being significantly higher than in the non-triggering situation. Additionally, the interaction was significant (*F*(1, 81) = 20.31, *p* < 0.001, η2 *p* = 0.200). A simple effect analysis indicated a significant difference (*p* < 0.001) in aggression between the presence and absence of trigger in the provocative situation, with participants in the triggering situation displaying stronger aggression. In the triggering situation, there was also a significant difference (*p* < 0.001) in aggression between the presence and absence of provocation, with participants showing stronger aggression. Conversely, in the non-provocative situation (*p* = 0.530) and non-triggering situation (*p* = 0.540), there were no significant differences. These findings suggest that participants exhibited the strongest aggression when both the provocative and triggering situations existed simultaneously (see Fig. 2).

### The mediating role of hostile attribution and the moderating role of the triggering situation

The correlation analysis revealed a significant positive correlation between anger, hostile attribution, and aggression (*ps* < 0.01), suggesting a relationship among these variables. To further explore the process of hostile attribution and aggression, a mediation analysis was conducted utilizing the bootstrap method. The results indicated that the indirect path of “anger → hostile attribution → aggression” was significant, with a 95% confidence interval (CI) of [0.21, 0.64], excluding 0. However, the direct path of “anger → aggression” was not significant, with a 95% CI of [–0.15, 0.24], including 0. This suggests that hostile attribution completely mediated the relationship between anger and aggression, confirming hypothesis *H*_1_.

**Fig. 3:**
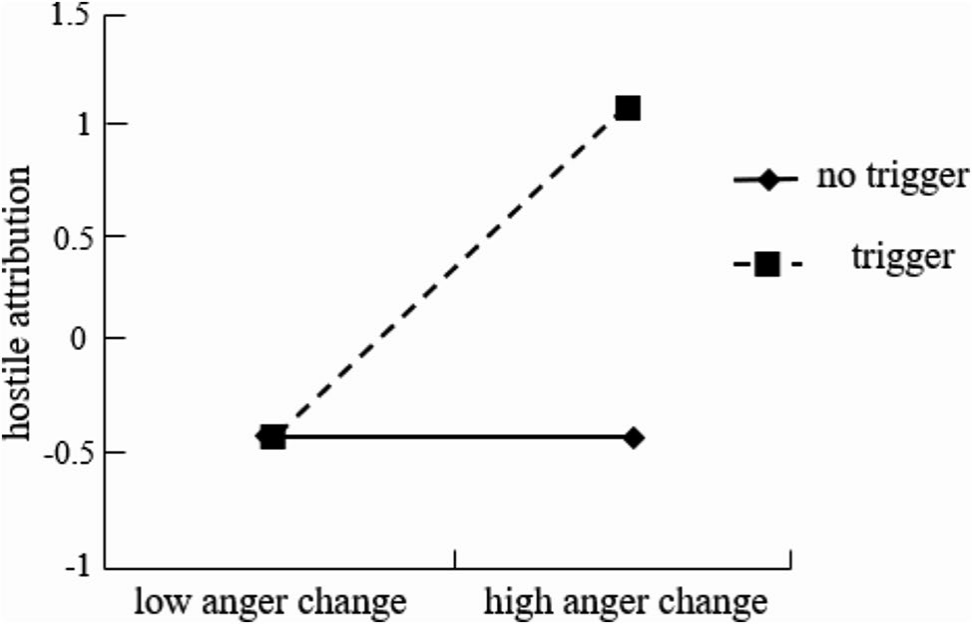
The moderating effect of the triggering situation

The experiment focused on the role of the triggering situation, which was a categorical variable (trigger or no trigger). To analyze its potential impact, it was converted into a dummy variable (trigger = 1, no trigger = 0). The subsequent analysis involved standardizing the variables to avoid multicollinearity and utilizing regression analysis to examine the moderating effect. In the first step, anger and the triggering situation were included in the regression equation. Then, the interaction terms between anger and the triggering situation were added. If the interaction term significantly predicted the dependent variable, it would indicate that the triggering situation had a substantial moderating effect on the relationship between anger and hostile attribution. The results demonstrated that both anger (β = 0.36, *p* < 0.001) and the triggering situation (β = 0.37, *p* < 0.001) significantly predicted hostile attribution. Furthermore, the interaction term between anger and the triggering situation was also significant (β = 0.36, *p* < 0.001), suggesting that the triggering situation indeed played a moderating role in influencing the link between anger and hostile attribution. To further analyze the moderating effect, a single slope analysis was conducted on the impact of anger on hostile attribution when the triggering situation was plus or minus one standard deviation. The analysis revealed that when there was a high triggering situation, increased anger significantly predicted higher levels of hostile attribution (*b*_simple_=0.72, *SE* = 0.09, *p* < 0.001). However, when there was a low triggering situation, the change in anger did not significantly predict hostile attribution (*b*_simple_=0.001, *SE* = 0.13, *p* = 0.970). This indicates that in the presence of a trigger, an individual’s hostile attribution to the trigger substantially increased with heightened anger. Conversely, when there was no trigger, changes in anger did not significantly affect hostile attribution (see Fig. 3).

#### Experiment 2: The moderating role of the trigger identity on TDA

##### Participants

In a university setting, a total of 85 undergraduate students (32 males and 53 females) were randomly chosen to participate in the experiment. However, three participants correctly guessed the intention of the experiment, leading to their invalidation. Therefore, the final number of participants included in the study was 82 (30 males and 52 females). The age range of the participants was between 18 and 22 years old, with an average age of 19.49 years and a standard deviation of 0.95.

#### Materials

In this study, the measurement of anger, hostile attribution, and aggression remained consistent with Experiment 1. The internal consistency coefficient for the Emotional State Self-Rating scale was 0.84, and for the Hostile Attribution Bias scale, it was 0.89.

### Group identity

In this study, the Group Identity Scale developed by Yzerbyt et al. [47] was utilized, consisting of 5 items such as “I am a member of our group” and “I have a very close relationship with the group members.” The scale used a 7-point scoring system, ranging from 1 = “completely disagree” to 7 = “completely agree.” The internal consistency coefficient of the scale was determined to be 0.71 in this study.

### Experimental design

A between-group design was employed, consisting of a 2 (trigger: yes, no) × 2 (trigger identity: in-group or out-group) design. The dependent variables measured in the experiment included anger, hostile attribution, and the score of aggression.

### Situation manipulation

#### Group formation

The minimal group paradigm, as described by Tajfel and Turner [48], was utilized to manipulate group formation. The participants were presented with works of two different painters, labeled painter A and painter B, in a total of 28 pairs. They were asked to select their favorite from each pair of works. Subsequently, the participants were divided into groups based on the painter they had preferred [49]. To strengthen group identification, the experimenter instructed the participants to complete a false personality questionnaire, informing them that individuals with similar responses would be placed into the same group.

### Provocative situation, triggering situation

The same as Experiment 1. Detailed materials can be found in the Supporting Information.

#### Procedure

In the experiment, participants were initially informed that they would be taking a personality test and were asked to select their preferred painting from each pair. They also completed a false personality questionnaire. The experiment was conducted with two groups—A and B, some of which included fake participants. Before the formal experiment, members of both groups were arranged to briefly meet, with the intention of making group B believe that the experiment was authentic. Communication content for the experiment was predetermined, and participants completed the Anger Self-Rating scale and Group Identity Scale. Subsequently, the same method from Experiment 1 was used to implement provocation (all participants were provoked) and induce anger in the participants. The triggers for the members of Group B came from members of the out-group (Group A) as well as other members of the in-group (Group B). Following the provocation, participants completed measurements of hostile attribution and indicated the level of reward the trigger should receive. After the experiment, participants were asked if they had guessed the purpose of the study. The experimenter explained the experiment’s content and provided psychological counseling to soothe the participants’ emotions.

## Results

### Effectiveness test of group formation and anger manipulation

The study utilized the scoring standard of Yzerbyt et al. [47], where the “4 (neutral)” scoring of the Group Identity scale was considered as the reference value. If the score exceeded “4,” a higher score indicated stronger in-group identity. The difference between the scores of in-group identity and “4” was compared. Results indicated that participants had a significantly stronger identity to the in-group (*M* = 5.31, *SD* = 0.77) compared to “4” (*t*_80_ = 15.40, *p* < 0.001, *d* = 1.70), demonstrating the successful formation of in-group and out-group identities through the minimal group paradigm. Moreover, participants’ anger after being provoked (*M* = 3.15, *SD* = 1.64) was significantly greater than before (*M* = 1.04, *SD* = 0.19; *t*_81_=–11.81, *p* < 0.001, *d* = 1.29), indicating the effective induction of anger during the experiment.

**Comparison of the differences between participants’ hostile attribution and the intensity of the attack under different experimental operations**.

The results of variance analysis are shown in Table 2.

**Table 2:** Anger, Hostile attribution, and TDA (*M ± SD*)

Situation type	Trigger identity	Hostile attribution	TDA
No Triggering Situation	in-group (*n* = 21)	9.05 ± 3.01	2.14 ± 0.79
	out-group (*n* = 21)	9.29 ± 3.89	2.24 ± 1.14
Triggering Situation	in-group (*n* = 18)	12.72 ± 6.14	2.61 ± 1.42
	out-group (*n* = 22)	20.23 ± 5.84	4.68 ± 1.29

**Fig. 4:**
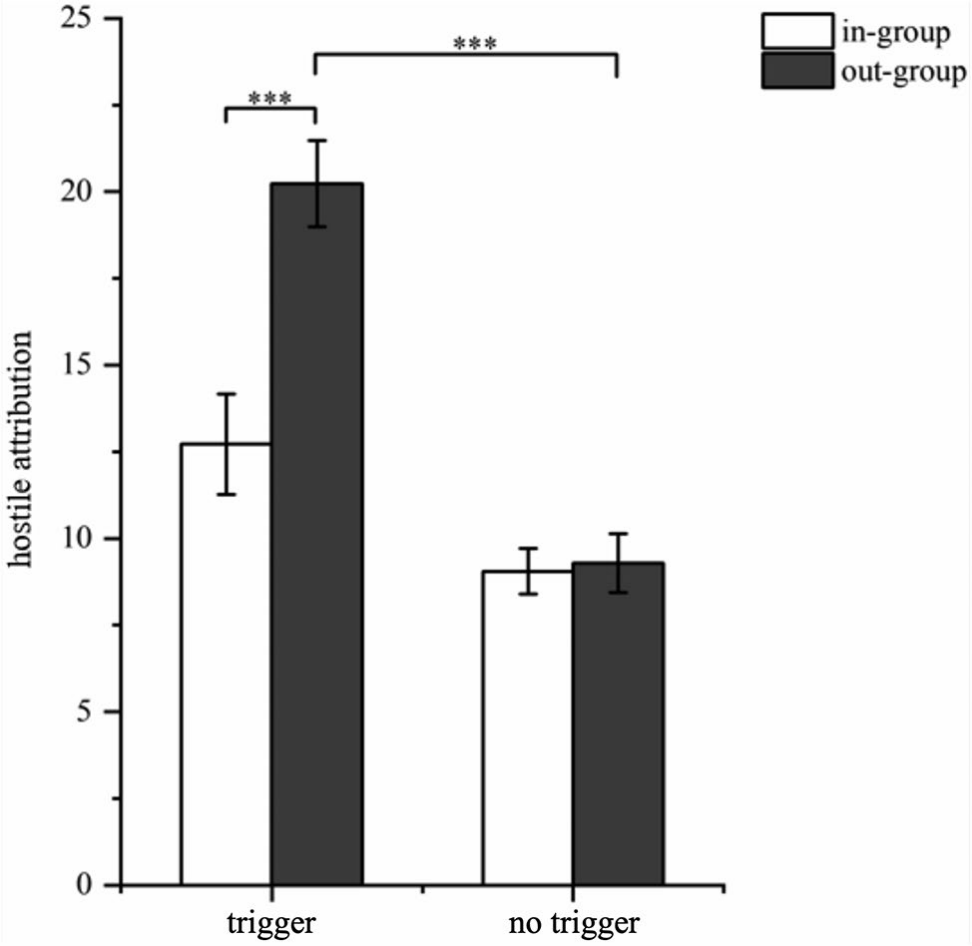
Individual’s hostile attribution towards in-group and out-group targets (*Note*: ^***^p < 0.001)

The analysis revealed that the main effect of the triggering situation was significant (*F*(1, 78) = 46.13, *p* < 0.001, η2 *p* = 0.372), indicating that the triggering situation resulted in significantly higher hostile attribution compared to the no-triggering situation. Additionally, the main effect of trigger identity was also significant (*F*(1, 78) = 12.95, *p* < 0.001, η2 *p* = 0.140), with the out-group eliciting significantly higher levels of hostile attribution than the in-group. The interaction between triggering situation and trigger identity was also significant (*F*(1, 78) = 11.40, *p* < 0.001, η2 *p* = 0.133). Further simple effect analysis indicated that there was no significant difference (*p* = 0.850) between the in-group and the out-group in the no-triggering situation. However, in the triggering situation, hostile attribution towards the out-group members was significantly higher than that towards in-group members (*p* < 0.001). Specifically, for in-group members, there was a significant difference between the triggering and the no-triggering situations (*p* < 0.05), with hostile attribution being significantly greater in the triggering situation. Similarly, for out-group members, the hostile attribution in the triggering situation was also significantly greater than in the no-triggering situation (*p* < 0.001; see Fig. 4). This suggests that regardless of whether the trigger was an in-group member or an out-group member, the triggering situation increased hostile attribution. Notably, hostile attribution was stronger when the trigger was an out-group member.

**Fig. 5:**
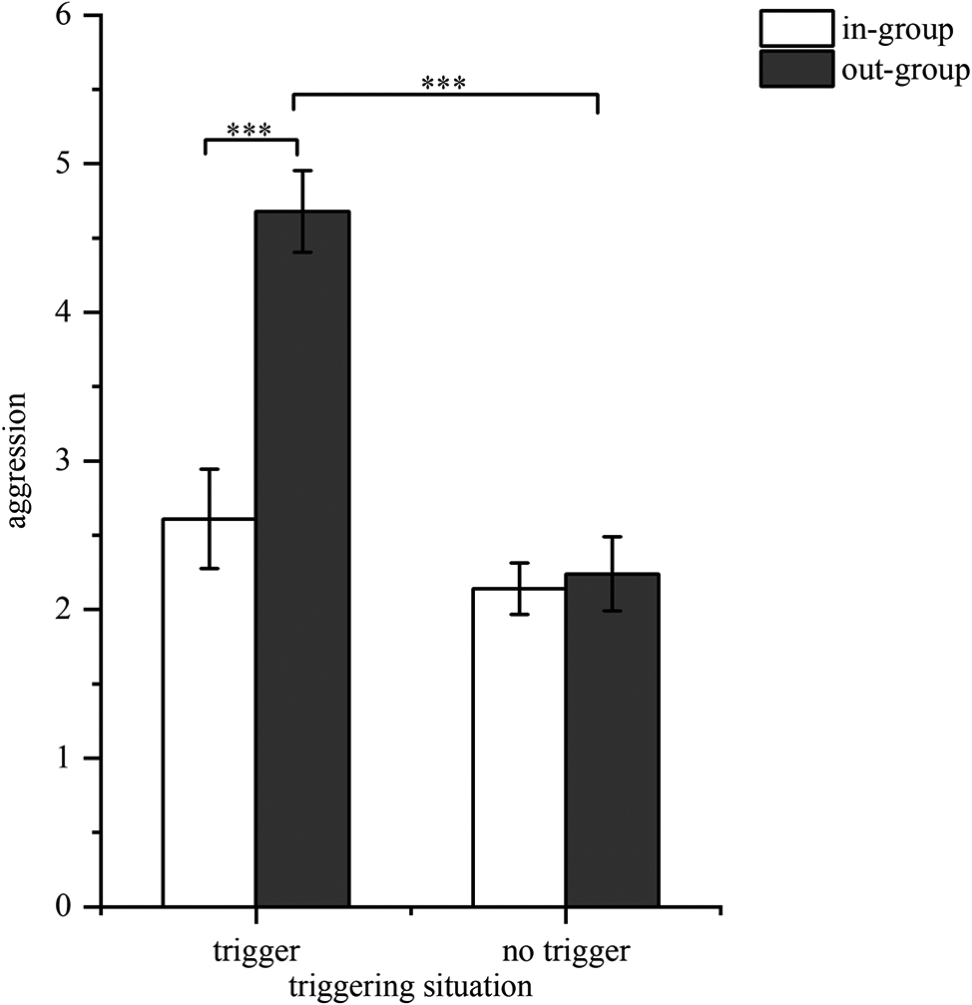
Individual’s aggression towards in-group and out-group targets (*Note*: ^***^p < 0.001)

The analysis of aggression revealed significant findings. The main effect of the triggering situation was significant (*F*(1, 78) = 33.90, *p* < 0.001, η2 *p* = 0.301), indicating that aggression in the triggering situation was significantly higher than in the no-triggering situation. Additionally, the main effect of trigger identity was also significant (*F*(1, 78) = 18.75, *p* < 0.001, η2 *p* = 0.192), with the out-group eliciting significantly higher levels of aggression than the in-group. The interaction between triggering situation and trigger identity was also significant (*F*(1, 78) = 15.60, *p* < 0.001, η2 *p* = 0.172). Further simple effect analysis indicated that there was no significant difference between the in-group and the out-group in the no-triggering situation (*p* = 0.790). However, in the triggering situation, participants’ aggression toward out-group members was significantly higher than toward in-group members (*p* < 0.001). Specifically, for in-group members, there was no significant difference between the triggering and the no-triggering situations (*p* = 0.200). For out-group members, aggression in the triggering situation was significantly greater than in the no-triggering situation (*p* < 0.001; see Fig. 5). Therefore, the results suggest that when a triggering situation occurs, participants tend to be more aggressive when the trigger is an out-group member.

### The mediating role of hostile attribution and the moderating role of triggering situation

**Fig. 6:**
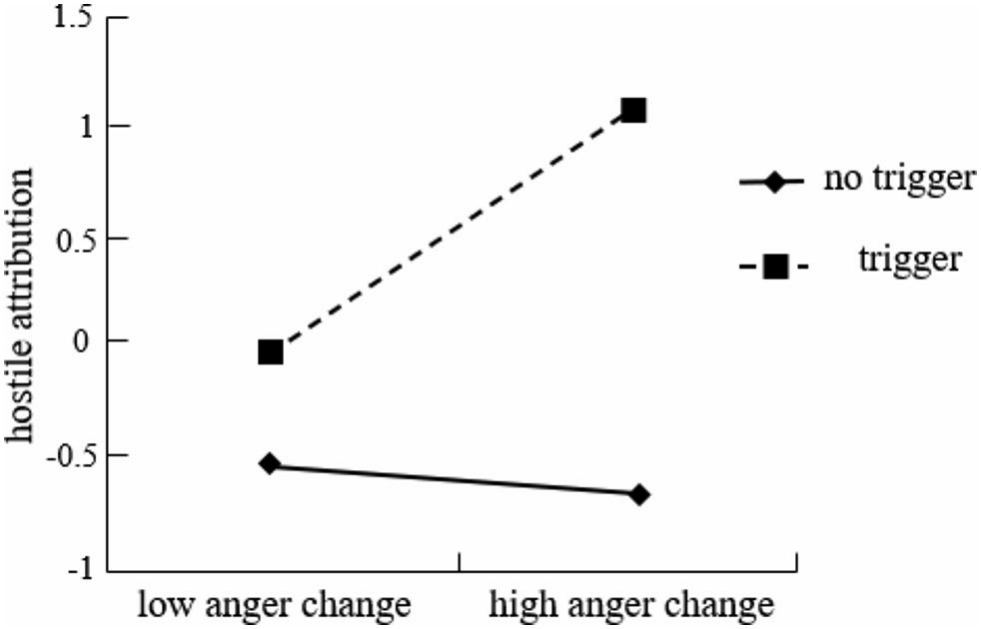
The moderating effect of the triggering situation

The results of the study revealed several significant associations and effects related to anger, hostile attribution, and aggression. There was a significant positive correlation between anger, hostile attribution, and aggression (*ps* < 0.01). The path of “anger → hostile attribution → aggression” was significant, indicating that hostile attribution completely mediated the relationship between anger and aggression. Furthermore, anger (β = 0.25, *p* < 0.01) and the triggering situation (β = 0.52, *p* < 0.001) were found to significantly predict hostile attribution. The interaction between anger and the triggering situation was significant (β = 0.28, *p* < 0.01), suggesting that the triggering situation played a moderating role in the influence of anger on hostile attribution. The single slope analysis demonstrated that when there was a high triggering situation, anger significantly predicted hostile attribution positively (*b*_simple_=0.53, *SE* = 0.14, *p* < 0.001). Conversely, when there was a low triggering situation, anger did not significantly predict hostile attribution (*b*_simple_=0.03, *SE* = 0.11, *p* = 0.762). These findings indicate that when there was a trigger, individuals’ hostile attribution to the trigger significantly increased with the increase in anger, whereas when there was no trigger, anger had no significant effect on hostile attribution. In conclusion, this study results highlight the complex interplay among anger, hostile attribution, and aggression, with the impact of triggering situations and the mediating role of hostile attribution providing important insights into the dynamics of these psychological processes (see Fig. 6).

### The moderating effect of the trigger identity

The study tested a moderated mediation model to further explore the influence of trigger identity. The trigger identity was converted into dummy variables, and all variables were standardized to avoid multicollinearity. The parameters of the three regression equations were tested according to the recommendations of Wen and Ye [50]. The results of the adjusted mediation model test showed that in the triggering situation, anger significantly predicted subsequent hostile attribution towards TDA. Therefore, only the triggering situation was considered in this analysis. The predictor variables were standardized in each equation, and the variance expansion factor of all predictors was lower than 1, indicating no multicollinearity concerns. In Eq. 1, anger significantly and positively predicted TDA, and the interaction between anger and trigger identity significantly predicted TDA. In Eqs. 2 and 3, the interaction between anger and trigger identity significantly predicted hostile attribution and TDA, and hostile attribution significantly and positively predicted TDA. These findings suggest that anger, hostile attribution, trigger identity, and aggression constitute a moderated mediation model. Hostile attribution mediated the relationship between anger and TDA, and trigger identity moderated the first half path and the direct path of the mediating process of “anger → hostile attribution → TDA.” This supports hypothesis *H*_2_, providing valuable insight into the complex interplay of these variables (see Table 3).

**Table 3:** Moderated mediation model test

Equation 1(Y: TDA)					Equation 2(M: hostile attribution)				Equation 3(Y: TDA)			
	*B*	*SE*	β	95%CI	*B*	*SE*	β	95%CI	*B*	*SE*	β	95%CI
*X*	0.23	0.10	0.23^*^	[0.03, 0.43]	0.26	0.10	0.26^*^	[0.05, 0.46]	0.08	0.09	0.08^*^	[0.09, 0.25]
*U* ^a^	0.35	0.10	0.35^**^	[0.15, 0.54]	0.29	0.10	0.29^**^	[0.08, 0.49]	0.19	0.09	0.19^*^	[0.02, 0.36]
*X*×*U*	0.22	0.10	0.22^*^	[0.02, 0.42]	0.15	0.11	0.15^*^	[0.05, 0.36]	0.13	0.09	0.13^*^	[0.02, 4.23]
*M*									0.55	0.10	0.55^***^	[0.36, 0.74]
*M*×*U*									0.04	0.10	0.03	[-0.16, 0.23]
*R* ^2^			0.22				0.17				0.47	
*F*			8.77^***^				6.44^**^				15.47^***^	

*NoteX* = anger, *U* = trigger identity, *M* = hostile attribution, *Y* = TDA; trigger identity is a dummy variable, in-group = 1, out-group = 2, and the 95% CI of all predictor variables were obtained by Bootstrap.

**Fig. 7:**
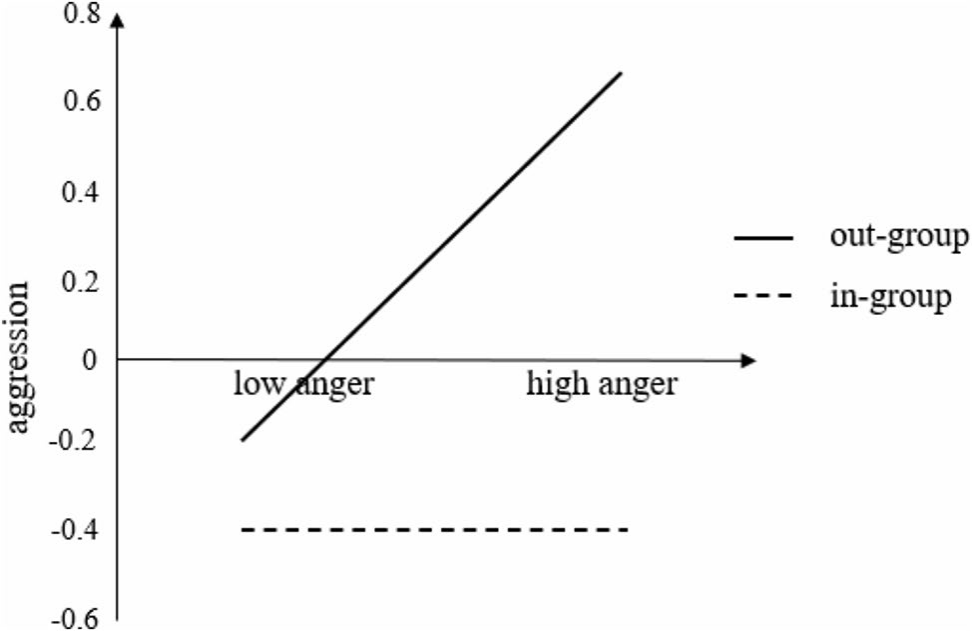
Moderating effect of the trigger identity

This study conducted a simple slope analysis to further examine the moderating role of trigger identity using the trigger identity plus or minus one standard deviation, focusing on the effect of TDA on anger. The results showed that when the trigger was an out-group member, anger significantly and positively predicted TDA (*b*_simple_=0.44, *SE* = 0.13, *p* < 0.001). However, when the trigger was an in-group member, anger did not significantly predict TDA (*b*_simple_=0.01, *SE* = 0.16, *p* = 0.350). This indicates that for the out-group trigger, as an individual’s anger increased, TDA also increased. Conversely, for the in-group trigger, there was no significant relationship between anger and TDA. These findings provide further support for the moderating role of trigger identity in the association between anger and TDA, shedding light on the differential effects of trigger identity on individuals’ responses to anger in relation to TDA (see Fig. 7).

## Discussion

Based on the “kicking the barking dog effect”, a psychological phenomenon where individuals redirect their frustration or anger towards a less powerful or vulnerable target, even if that target is not the original source of their negative emotions, this study conducted three experiments and obtained meaningful results.

### Internal process of TDA

From the standpoint of the continuity of emotion and cognition-behavior, this study sought to explore the internal process of TDA from both individual and group perspectives. The preliminary experiment conducted a verification of the influence of emotion on cognition and found that anger leads to stronger hostile attribution in subsequent ambiguous situations, which aligns with previous research findings [51]. The influence of emotion on cognition serves as a bridge connecting the preceding and subsequent triggering situations in TDA. Experiment 1 provided a comprehensive examination of the prior provocative situation, the emotional state it provoked, the subsequent triggering situation, the individual’s cognitive processing of it, and the expression of the individual’s aggressive behavior. The results indicated that the anger provoked by the initial situation influenced TDA by shaping the individual’s hostile attribution toward the trigger. The key to the occurrence of TDA is the influence of the previous state on subsequent cognitive processing. The previous provocative situation and the anger that it incites are the premise for TDA, while the subsequent triggering situation is the key to the occurrence of aggressive behavior. Experiment 2 studied TDA under a group framework. The results found that hostile attribution played a mediating role between anger and aggression, which further verified that TDA originated from the state caused by the provocation, and this affected the individual’s cognitive processing of the triggering situation. Experiment 2 also revealed that when the trigger was an in-group member, individuals still exhibited hostile attribution towards it; however, aggression was not displayed. This suggests that there are additional influencing factors between hostile attribution and the expression of aggression, which warrant further investigation in a follow-up study. Given that people cannot avoid previous provocative situations in real life, strategies can be adopted to reduce the occurrence of TDA. Firstly, emotions provoked by the provocation should be dissipated as quickly as possible, mitigating the premise for TDA. Additionally, strategies such as cognitive reappraisal can weaken hostile attribution towards the trigger. Cognitive reappraisal is an advanced attention strategy that modifies the emotional response by altering the understanding of emotional events [52]. Through cognitive reappraisal, individuals can develop a new interpretation of events, thereby reducing hostile explanations and decreasing the likelihood of aggressive expression.

#### The role of the trigger identity in the process of TDA

In real-life intergroup violent conflicts, direct violent confrontations between groups are not common. Instead, conflicts typically arise from the diffusion and escalation of aggressive behaviors between members of the two groups, involving the spread of aggression from individuals to multiple individuals. However, existing research on this issue is not sufficiently comprehensive. For example, the general aggression model primarily focuses on the origin and progression of an individual’s aggressive behavior [11]. In contrast, TDA involves the transfer of attack targets, offering a unique perspective for better understanding the escalation of intergroup violent conflicts. Experiment 2 delved into the generation process of TDA within a group framework and thoroughly considered the role of trigger identity. The results indicated that individuals exhibited stronger hostile attribution and aggressive behavior toward out-group triggers, aligning with previous research findings. According to social identity theory, individuals tend to favor the in-group while rejecting the out-group to enhance their self-esteem [49]. Consequently, when out-group members display negative behaviors, individuals are inclined to make stable and internal attributions and exhibit stronger aggressive behaviors [5]. As a result, TDA may contribute to the escalation of intergroup violent conflicts through two mechanisms. Firstly, when an individual is provoked and an out-group member triggers them, the individual is likely to direct their anger toward the out-group member, posing a latent risk for intergroup violent conflicts. Secondly, if an individual is provoked by an out-group member, they are more likely to displace aggression by directing it toward other out-group members, leading to the spread of aggressive behavior from two individuals to three or more. If the victim of displaced aggression also adopts the TDA behavioral pattern, the attack could expand to involve even more individuals, ultimately fostering violent conflicts between the two groups. Given that people often cannot avoid the initial provocative situation and subsequent triggering events, the question arises: how can conflict escalation be prevented? Experiment 2 revealed that within the group framework, anger stemming from provocation also influences subsequent aggressive behavior through hostile attribution. Similar to the individual framework, dissipating the anger provoked by the situation as quickly as possible can help mitigate aggression. Additionally, Experiment 2 demonstrated that group identity moderates the impact of hostile attribution on aggression. Specifically, when the trigger is an in-group member, the individual’s aggressive behavior increases with a rise in hostile attribution. Conversely, if the trigger is an out-group member, strong aggressive behavior may still be displayed even in the absence of intense hostile attribution. This suggests that solely reducing hostile attribution may not be an effective approach to mitigating conflict escalation within the group framework. However, if hostile attribution is reduced by altering group identity awareness (e.g., shifting from small group identification to large group identification), it may significantly decrease the likelihood of violent conflicts and prevent the expansion of conflicts from individuals to groups.

### Research significance and prospects

This study discovered that the emergence of trigger substitution is instigated by anger, which in turn stimulates individuals’ hostile attribution, leading to the manifestation of TDA. It highlights the complexity and significance of the TDA phenomenon. Furthermore, the research also indicated, to a certain extent, that individual aggression is a pivotal factor in group conflict. The aggressive behavior observed in group conflict may result from the transfer of displaced aggression between in-group and out-group members. Identifying targets as in-group or out-group members manifests TDA as a phenomenon of displaced retaliation between groups. Given that individuals have distinct group affiliations, conflicts between individuals from different groups can give rise to a dynamic process of intergroup conflict. The findings of this study hold practical guidance value. Firstly, the study ascertained that hostile attribution played a mediating role in the influence of anger resulting from a provocative situation on TDA. Therefore, it is possible to reduce the occurrence of TDA by mitigating an individual’s anger, or by redirecting their attention to free them from the entrapment of emotional thinking. Cognitive reappraisal can also be employed to weaken hostile attribution towards the trigger [52]. Secondly, the identity of in-group and out-group targets moderates the impact of hostile attribution on aggressive behavior. This suggests that altering perceptions of group identity (such as elevating the perception from a small group to a larger group) can diminish hostile attribution, thereby reducing the likelihood of violent conflict escalation from individuals to groups. Thirdly, the conclusions of this study offer practical guidance for areas such as organizational behavior and educational contexts. For instance, leaders in organizations can work to diminish employees’ hostile attributions to mitigate TDA.

This study has a number of limitations that should be considered. Firstly, the study assumes that emotion (anger) influences cognition (hostile attribution), but the relationship between emotion and cognition is not one-way. Individuals who frequently demonstrate hostile attribution may also experience anger more often [53]. Conversely, reducing hostile attribution through intervention can effectively decrease an individual’s experience of anger [54]. The occurrence of TDA is likely to result from the interaction between emotion and cognition, and future research should explore the impact of this two-way relationship on TDA. Secondly, the use of minimum group paradigm manipulation to establish in-group and out-group dynamics may aid in standardizing experimental research operations. However, compared to real-life groups, the experimental groups lacked the involvement and commitment typically found in authentic group settings, thus diminishing ecological validity. Subsequent research should focus on different real-life groups (such as various fan groups, religious groups, etc.) to investigate the generation process of TDA. Thirdly, since the participants in this study were college students, the conclusions drawn are limited to this specific population and cannot be generalized to a broader group. Future research could appropriately expand the participant scope, which would not only verify the conclusions of this study but also extend the findings to a wider audience.

## Conclusion

The conclusions of the current study are as follows. First, when provocation and triggering occurred simultaneously, individuals displayed stronger hostile attribution and aggression toward the trigger. Second, hostile attribution played a completely mediating role, and the triggering situation acted as a moderator in the relationship between anger and TDA. When there was a triggering situation, individuals exhibited stronger hostile attribution as anger increased. Conversely, when there was no triggering situation, changes in anger had no significant effect. Third, trigger identity played a moderating role in the process of “anger → hostile attribution → TDA” in the triggering situation. For an in-group trigger, individuals made stronger hostile attributions to the out-group and subsequently engaged in TDA. However, for an out-group trigger, there was no significant effect.

## Electronic Supplementary Material

Below is the link to the electronic supplementary material.

Supplementary Material 1

## Data Availability

Data sharing not applicable to this article as no datasets were generated or analyzed during the current study.
