# Correction: Effects of anger and trigger identity on triggered displaced aggression among college students: based on the “kicking the barking dog effect”

**DOI:** 10.1186/s40359-024-02216-4

**Published:** 2024-11-25

**Authors:** Shen Liu, Wenxiu Li, Xinwei Hong, Minghua Song, Feng Liu, Zhibin Guo, Lin Zhang

**Affiliations:** 1 Department of Psychology, School of Humanities and Social Sciences, Anhui Agricultural University, No. 130 Changjiang Road(W), Shushan District, Hefei, Anhui 230036 China; 2 Department of Psychology, Renmin University of China, Beijing, China; 3 Department and Institute of Psychology, Ningbo University, No. 616 Fenghua Road, Jiangbei District, Ningbo, Zhejiang 315211 China; 4 Mental Health Education Guidance Center, Huzhou University, Huzhou, Zhejiang China; 5 Xiamen University Tan Kan Kee College, Zhangzhou, Fujian China

**Correction: BMC Psychology (2024) 12:641**

10.1186/s40359-024-02118-5

Following publication of the original article, it came to the journal’s attention that the affiliations of the first author, Shen Liu, were incorrect: the author had been affiliated with affiliations 1 and 5, whereas they should just be affiliated with affiliation 1. The article has since been corrected. 

