# Supplementary material for: Effects of anger and trigger identity on triggered displaced aggression among college students: based on the “kicking the barking dog effect”
Source: BMC Psychol. 2024 Nov 8;12:641. doi: 10.1186/s40359-024-02118-5 (PMC11545097; doi:10.1186/s40359-024-02118-5)
Supplement: Supplementary file 1 — Supplementary Material 1 [file 40359_2024_2118_MOESM1_ESM.doc]

**Supporting Information**

**Preliminary experiment**

**Emotional State Self-Rating scale**

Sex: ○ Male ○ Female Age: Family of origin: ○ City ○ Village

Single child: ○ yes ○ no

Please choose between 1 and 7, according to how you feel at the moment, where 1 means “not at all” and 7 means “very strong.”

The intensity of the feeling increases with the number from left to right.

|  | 1 | 2 | 3 | 4 | 5 | 6 | 7 |
| --- | --- | --- | --- | --- | --- | --- | --- |
| hate |  |  |  |  |  |  |  |
| pain |  |  |  |  |  |  |  |
| excitement |  |  |  |  |  |  |  |
| nervousness |  |  |  |  |  |  |  |
| fear |  |  |  |  |  |  |  |
| anger |  |  |  |  |  |  |  |
| happiness |  |  |  |  |  |  |  |

**Anger activation material**

**Control group**: Please carefully recall a description about a product or an explanation of a word in a book, and answer the following questions based on your own memories.

1. When did you see the information (product description or word explanation)?—it can be the approximate time.

.

2. Where did you see the information?

.

3. Briefly describe what you recall.

.

**Experimental group:** Please carefully recall a recent experience that made you feel angry (in this experience, you were so angry that you wanted to vent your anger, and even when you think about it now, you still feel angry). Please hold this experience in your mind for one minute and answer the following questions based on your memory.

1. When did the experience happen (it can be approximate time)?

.

2. Where did the experience happen?

.

3. Briefly describe the experience.

.

**Hostile attribution and aggression**

Please read the following stories carefully, imagine they happened to you, and answer every question after the story. There are no right or wrong answers; please choose according to your first image.

1. Imagine you are walking on the school corridor, and two classmates are coming toward you. When you pass by them, they look at you, whisper to each other, and laugh.

(1) Why do you think they laughed? (1 means “impossible” and 7 means “very possible,” increasing step by step)

|  | 1 | 2 | 3 | 4 | 5 | 6 | 7 |
| --- | --- | --- | --- | --- | --- | --- | --- |
| ① They were laughing at me. |  |  |  |  |  |  |  |
| ② They were telling jokes. |  |  |  |  |  |  |  |
| ③ There are very happy today. |  |  |  |  |  |  |  |
| ④ They tried to make me angry. |  |  |  |  |  |  |  |

(2) Do you think the two classmates’ behavior is benign or malicious? (the larger the number, the higher the degree of maliciousness)

Completely benign ○ 1 ○ 2 ○ 3 ○ 4 ○ 5 ○ 6 ○ 7 Completely malicious

(3) How much do you want to be angry with these two classmates (the larger the number, the higher the degree)

Not at all ○ 1 ○ 2 ○ 3 ○ 4 ○ 5 ○ 6 ○ 7 A lot

2 Imagine you have arranged with a classmate to go on a short trip on the weekend. You are very excited about it and have been looking forward to the weekend. However, on Friday night, your classmate suddenly tells you that he/she has made plans with someone else and will not go on a trip with you.

(1) Why doesn’t your classmate want to go on a trip with you? (1 means “impossible” and 7 means “very possible,” increasing step by step)

|  | 1 | 2 | 3 | 4 | 5 | 6 | 7 |
| --- | --- | --- | --- | --- | --- | --- | --- |
| 1. He/she does not want to be with me. |  |  |  |  |  |  |  |
| 1. He/she prefers to do other things. |  |  |  |  |  |  |  |
| 1. He/she has forgotten about our plans. |  |  |  |  |  |  |  |
| 1. He/she does not care about me. |  |  |  |  |  |  |  |

(2) Do you think the classmate’s behavior is benign or malicious? (the larger the number, the higher the degree of maliciousness)

Completely benign ○ 1 ○ 2 ○ 3 ○ 4 ○ 5 ○ 6 ○ 7 Completely malicious

(3) How much do you want to be angry with this classmate (the larger the number, the higher the degree)

Not at all ○ 1 ○ 2 ○ 3 ○ 4 ○ 5 ○ 6 ○ 7 A lot

3 Imagine you are looking for a place to sit down for lunch. You see some people you know sitting on the opposite table. They are talking and laughing with each other. They are very happy. When you walk up to them and sit down beside them, they stop talking, and no one talks to you.

(1) Why did they stop talking when you sat down? (1 means “impossible” and 7 means “very possible,” increasing step by step)

|  | 1 | 2 | 3 | 4 | 5 | 6 | 7 |
| --- | --- | --- | --- | --- | --- | --- | --- |
| ① They were waiting for me to speak first. |  |  |  |  |  |  |  |
| ② They did not want to talk to me. |  |  |  |  |  |  |  |
| ③ They were saying bad things about me before I got there. |  |  |  |  |  |  |  |
| ④ They happened to have just finished conversation. |  |  |  |  |  |  |  |

(2) Do you think these classmates’ behavior is benign or malicious? (the larger the number, the higher the degree of maliciousness)

Completely benign ○ 1 ○ 2 ○ 3 ○ 4 ○ 5 ○ 6 ○ 7 Completely malicious

(3) How much do you want to be angry with these classmates (the larger the number, the higher the degree)

Not at all ○ 1 ○ 2 ○ 3 ○ 4 ○ 5 ○ 6 ○ 7 A lot

4 Imagine you are eating in the dining hall, when two classmates come in, chatting to each other. You hear one of them inviting the other to his/her birthday party, and saying that there will be many classmates at the party, but you have not received any invitation.

(1) Why did not the classmate invite you to the party? (1 means “impossible” and 7 means “very possible,” increasing step by step)

|  | 1 | 2 | 3 | 4 | 5 | 6 | 7 |
| --- | --- | --- | --- | --- | --- | --- | --- |
| 1. He/she does not want me to participate. |  |  |  |  |  |  |  |
| 1. He/she has not had a chance to invite me yet. |  |  |  |  |  |  |  |
| 1. He/she wants to get back at me for something. |  |  |  |  |  |  |  |
| 1. He/she will invite me to attend later. |  |  |  |  |  |  |  |

(2) Do you think the classmate’s behavior is benign or malicious? (the larger the number, the higher the degree of maliciousness)

Completely benign ○ 1 ○ 2 ○ 3 ○ 4 ○ 5 ○ 6 ○ 7 Completely malicious

(3) You do not want to pay any more attention to that classmate. (the larger the number, the higher the degree)

Not at all ○ 1 ○ 2 ○ 3 ○ 4 ○ 5 ○ 6 ○ 7 A lot

5 Imagine you are walking on campus and see two of your classmates. You walk towards them and greet them. However, they treat you as if you do not exist, and do not say anything to you. Then, they start chatting to each other (you cannot hear) and walk the other way.

(1) Why did not these two classmates say hello to you? (1 means “impossible” and 7 means “very possible,” increasing step by step)

|  | 1 | 2 | 3 | 4 | 5 | 6 | 7 |
| --- | --- | --- | --- | --- | --- | --- | --- |
| ① They did not see me standing there. |  |  |  |  |  |  |  |
| ② They did not hear me greeting them. |  |  |  |  |  |  |  |
| ③ They are angry with me because of something. |  |  |  |  |  |  |  |
| ④ They do not like me. |  |  |  |  |  |  |  |

(2) Do you think the two classmates’ behavior is benign or malicious? (the larger the number, the higher the degree of maliciousness)

Completely benign ○ 1 ○ 2 ○ 3 ○ 4 ○ 5 ○ 6 ○ 7 Completely malicious

(3) How much do you want to be angry with these two classmates? (the larger the number, the higher the degree)

Not at all ○ 1 ○ 2 ○ 3 ○ 4 ○ 5 ○ 6 ○ 7 A lot

6 Imagine that one day, when you leave a shop in downtown, the alarm suddenly goes off. There are other people who were leaving the store at the same time as you, but the security guard only stops you and insists on checking your bag with a very unfriendly attitude. Of course, the result of the inspection is that you did not take anything. At this time, he had to let you go and said that such misunderstandings often happen in that store.

(1) Why did the security guard stop you alone? (1 means “impossible” and 7 means “very possible,” increasing step by step)

|  | 1 | 2 | 3 | 4 | 5 | 6 | 7 |
| --- | --- | --- | --- | --- | --- | --- | --- |
| ① The security guard did not like me and was deliberately targeting me. |  |  |  |  |  |  |  |
| ② The security guard was doing his job as usual. |  |  |  |  |  |  |  |
| ③ The security guard picked a random person and it happened to be me. |  |  |  |  |  |  |  |
| ④ The security guard thought I was a student and easy to bully. |  |  |  |  |  |  |  |

(2) Do you think the security guard’s behavior is benign or malicious? (the larger the number, the higher the degree of maliciousness)

Completely benign ○ 1 ○ 2 ○ 3 ○ 4 ○ 5 ○ 6 ○ 7 Completely malicious

(3) How much do you want to be angry with the security guard (the larger the number, the higher the degree)

Not at all ○ 1 ○ 2 ○ 3 ○ 4 ○ 5 ○ 6 ○ 7 A lot

**Experiment 1**

**Basic information and Emotional State Self-Rating scale**

Hello! Please fill in truthfully according to your current situation. We will keep your personal information confidential. Thank you for your support.

Number: . Grade . Age .

Your sex: ○ Male ○Female Family of origin: ○ City ○ Village

Single child: ○ yes ○ no

Please choose between 1–7 according to what you feel at the moment. Each number represents the degree of your emotion at the moment, where 1 means “not at all,” and 7 means “very strong.”

The intensity of the feeling increases with the number from left to right.

|  | 1 | 2 | 3 | 4 | 5 | 6 | 7 |
| --- | --- | --- | --- | --- | --- | --- | --- |
| hate |  |  |  |  |  |  |  |
| pain |  |  |  |  |  |  |  |
| excitement |  |  |  |  |  |  |  |
| nervousness |  |  |  |  |  |  |  |
| fear |  |  |  |  |  |  |  |
| anger |  |  |  |  |  |  |  |
| happiness |  |  |  |  |  |  |  |

**Provocative material**

**Content:**

Suppose you are a salesperson in a bread company. The company sends you to a remote area to destroy a truckload of expired bread (although the bread has expired, it will not kill you or harm your health). On the way, you meet a group of hungry refugees blocking the way, because they firmly believe that there is something to eat in your truck. At the same time, reporters are covering the movements of arriving refugees. The refugees must solve the problem of hunger; the reporters want to report the facts; and you must destroy bread.

**Problems:**

Now you are required to solve the hunger problem of the refugees and let them eat the expired bread (not fatal and harmless to their health) to destroy the bread, but you cannot let the reporters report the fact. How will you deal with it? (Please complete within 5 minutes)

Notes: 1. The expired bread will not be harmful; 2. Do not bribe reporters; 3. Do not damage the company’s image.

**Feedback from the provocative group**

Your answer is not good enough. Have you considered the interests of the company, the purpose of the reporters, and the suffering of the refugees? I thought the people who came to participate in this experiment would at least be on the same level...but your answer...ho...

**Feedback from the no provocative group**

The total score is 10, and you got 6.7 points, which is above the average level.

**Triggering material**

Please list as many qualities as possible of an excellent astronaut within 3 minutes, and briefly explain the reason behind your choices. We will rate you based on creativity, quality, effort, diversity, and rationality.

**Feedback from the triggering group**

| 1.creativity | not good at all | 1 | 2 | 3 | 4 | 5 | 6 | 7 | very good | 1 |
| --- | --- | --- | --- | --- | --- | --- | --- | --- | --- | --- |
| 2.quality | not good at all | 1 | 2 | 3 | 4 | 5 | 6 | 7 | very good | 2 |
| 3.effort | not good at all | 1 | 2 | 3 | 4 | 5 | 6 | 7 | very good | 3 |
| 4.diversity | not good at all | 1 | 2 | 3 | 4 | 5 | 6 | 7 | very good | 1 |
| 5.rationality | not good at all | 1 | 2 | 3 | 4 | 5 | 6 | 7 | very good | 3 |
| 6.total | not good at all | 1 | 2 | 3 | 4 | 5 | 6 | 7 | very good | 2 |

*NOTE*: The red numbers in the table are the scores given to the participants, the same below.

Comment: In my opinion, the completion is not good, I think that, as a person who has the ability to think independently, you should perform better.

**Feedback from the no triggering group**

| 1.creativity | not good at all | 1 | 2 | 3 | 4 | 5 | 6 | 7 | very good | 6 |
| --- | --- | --- | --- | --- | --- | --- | --- | --- | --- | --- |
| 2.quality | not good at all | 1 | 2 | 3 | 4 | 5 | 6 | 7 | very good | 5 |
| 3. effect | not good at all | 1 | 2 | 3 | 4 | 5 | 6 | 7 | very good | 6 |
| 4. diversity | not good at all | 1 | 2 | 3 | 4 | 5 | 6 | 7 | very good | 5 |
| 5. rationality | not good at all | 1 | 2 | 3 | 4 | 5 | 6 | 7 | very good | 5 |
| 6.total | not good at all | 1 | 2 | 3 | 4 | 5 | 6 | 7 | very good | 5 |

Comment: It is okay to complete in a limited time.

**Hostile attribution and aggression**

Number: .

1. Please answer the following questions according to your first impression based on assistant B’s response.

|  | completely inconsistent | moderately inconsistent | slightly inconsistent | uncertain | slightly inconsistent | moderately consistent | completely consistent |
| --- | --- | --- | --- | --- | --- | --- | --- |
| I am pleased with assistant B’s performance. |  |  |  |  |  |  |  |
| Assistant B did this to me on purpose. |  |  |  |  |  |  |  |
| Assistant B is unreasonable. |  |  |  |  |  |  |  |
| Assistant B is hostile to me. |  |  |  |  |  |  |  |
| Assistant B is biased against me. |  |  |  |  |  |  |  |

2. Please decide the level of reward that assistant B should receive. The higher the level, the higher the reward.

Low ○ 1 ○ 2 ○ 3 ○ 4 ○ 5 ○ 6 ○ 7 High

**Experiment 2**

**The simplest grouping task**

There is no right or wrong option; please choose according to your first impression.

Number: .

1. Please choose the picture that you prefer.


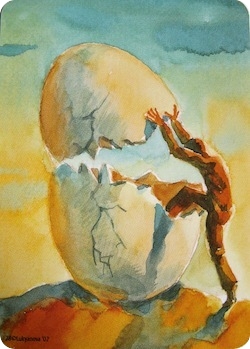

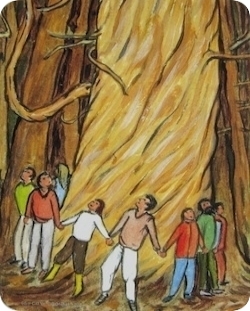


○ 1 ○ 2

2. Please select the picture that best represents how you were as a teenager.


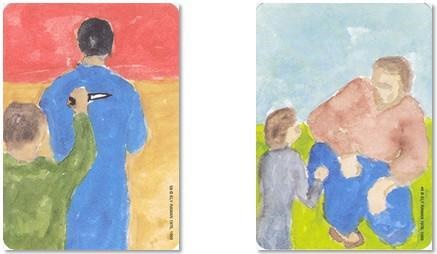


○ 1 ○ 2

3. Please choose the picture that best matches your current interpersonal situation.


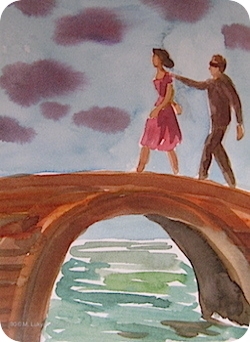

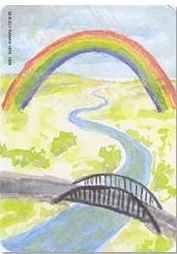


○ 1 ○ 2

4. Do you have to stop and think before you do anything?

○ Yes ○ No

5. Can you usually enjoy yourself in a lively party?

○ Yes ○ No

6. Did you spend your childhood doing whatever you were told, without complaining?

○ Yes ○ No

7. Do you easily understand the grievances of others when they complain to you?

○ Yes ○ No

8. Do you get excited easily?

○ Yes ○ No

9. Do you usually take the initiative when making new friends?

○ Yes ○ No

10. Do you often feel “bored”?

○ Yes ○ No

**Group identity task**

Hello! Please fill in truthfully, according to your current situation. We will keep your personal information confidential. Thank you for your support.

Number: . Grade . Age . Grouping .

The name of the student you are communicating with in this section: .

Sex: ○ Male ○ Female Family of origin: ○ city ○ village

Single child: ○ yes ○ no

1. Please answer the following questions based on your true feelings and attitudes.

|  | completely disagree | moderately disagree | slightly disagree | uncertain | slightly agree | moderately agree | completely agree |
| --- | --- | --- | --- | --- | --- | --- | --- |
| I am part of our group. |  |  |  |  |  |  |  |
| I am glad to be in this group. |  |  |  |  |  |  |  |
| I am happy to finish the task with the other members of the group. |  |  |  |  |  |  |  |

2. Please answer with your first instinct.

|  | extremely different | very different | slightly different | uncertain | slightly similar | very similar | extremely similar |
| --- | --- | --- | --- | --- | --- | --- | --- |
| You feel a similarity in identity or personality and abilities with other members of your group. |  |  |  |  |  |  |  |

3. Please answer with your first instinct.

|  | completely unconnected | very distant | slightly distant | uncertain | slightly close | very close | extremely close |
| --- | --- | --- | --- | --- | --- | --- | --- |
| How do you feel toward the group members you just met? |  |  |  |  |  |  |  |

**Hostile attribution and aggression**

Number: Grouping .

1. The cooperative members of the third part belong to the same group as me.

○ Yes ○ No

2. The name of the member you are working with in this section (third part): .

3. Please answer the following questions according to your first feelings, based on the reactions of the partner in the third part.

|  | completely inconsistent | moderately inconsistent | slightly inconsistent | uncertain | slightly consistent | moderately consistent | completely consistent |
| --- | --- | --- | --- | --- | --- | --- | --- |
| I am satisfied with the performance of the member of the third part. |  |  |  |  |  |  |  |
| The third part of the cooperation member to me is deliberate. |  |  |  |  |  |  |  |
| The third part of the cooperation member is unreasonable. |  |  |  |  |  |  |  |
| The third part of the cooperation member is hostile to me. |  |  |  |  |  |  |  |
| The third part of the cooperation member is biased against me. |  |  |  |  |  |  |  |

4. Please decide the level of reward that the third part of the cooperation member should receive. The higher the level, the more reward assistant B will receive.

few ○ 1 ○ 2 ○ 3 ○ 4 ○ 5 ○ 6 ○ 7 more
